# Optimization of Fiber Bragg Gratings Inscribed in Thin Films Deposited on D-Shaped Optical Fibers

**DOI:** 10.3390/s21124056

**Published:** 2021-06-12

**Authors:** José Javier Imas, Carlos R. Zamarreño, Ignacio del Villar, Ignacio R. Matías

**Affiliations:** 1 Electrical, Electronics and Communications Engineering Department, Public University of Navarra, 31006 Pamplona, Spain; josejavier.imas@unavarra.es (J.J.I.); ignacio.delvillar@unavarra.es (I.d.V.); natxo@unavarra.es (I.R.M.); 2 Institute of Smart Cities (ISC), Public University of Navarra, 31006 Pamplona, Spain

**Keywords:** fiber Bragg grating, D-shaped optical fiber, sensitivity, FWHM, FOM

## Abstract

A fiber Bragg grating patterned on a SnO_2_ thin film deposited on the flat surface of a D-shaped polished optical fiber is studied in this work. The fabrication parameters of this structure were optimized to achieve a trade-off among reflected power, full width half maximum (FWHM), sensitivity to the surrounding refractive index (SRI), and figure of merit (FOM). In the first place, the influence of the thin film thickness, the cladding thickness between the core and the flat surface of the D-shaped fiber (neck), and the length of the D-shaped zone over the reflected power and the FWHM were assessed. Reflected peak powers in the range from −2 dB to −10 dB can be easily achieved with FWHM below 100 pm. In the second place, the sensitivity to the SRI, the FWHM, and the FOM were analyzed for variations of the SRI in the 1.33–1.4 range, the neck, and the thin-film thickness. The best sensitivities theoretically achieved for this device are next to 40 nm/RIU, while the best FOM has a value of 114 RIU^−1^.

## 1. Introduction

Fiber Bragg gratings (FBG) consist of periodic perturbations of the refractive index along a fiber (generally, a single mode fiber) formed by exposure of the core to an intense optical interference pattern [1,2]. These perturbations lead to the generation of resonances in the optical transmission and reflection spectrum by coupling of light from the core mode to another co-propagating or counter-propagating mode that might be guided in the core or in the cladding of the optical fiber [3]. 

Fiber Bragg gratings can be classified into two groups depending on the grating period length: short-period fiber Bragg gratings, which are usually what is referred to by the name fiber Bragg gratings (FBGs), and long-period fiber gratings (LPGs). In the case of the former, the coupling is between the core mode and counter-propagating modes, whereas in the case of the latter, the coupling occurs between the core mode and a co-propagating cladding mode [3]. Among FBGs, it can be distinguished between devices in which light is coupled between the core mode and the counter propagating core mode, and those where a coupling between the core mode and the counter-propagating cladding modes is enhanced by employing a tilted fiber Bragg grating (TFBG) [4].

FBGs are the most commercially employed type of fiber gratings, thanks to their role in structural health monitoring. Several different gratings can be inscribed in an optical fiber, enabling to measure strain and temperature in different points of a structure, such as aircrafts, tunnels, dams, etc. by using wavelength multiplexing of the different resonance wavelengths of the FBGs [2].

However, FBGs are insensitive to the changes of the refractive index of the surrounding medium (SRI), as light coupling only takes place between core modes, which are not affected by the medium thanks to the fiber cladding. In order to make FBGs sensitive to the SRI, it is necessary to reduce the cladding thickness around the grating region via an etching process, resulting in an eFBG, also known as thinned or reduced cladding FBG [5].

Fiber gratings are also combined with surface plasmon resonances (SPR) as they improve the SPR excitation efficiency, enabling the development of sensing platforms for the detection of gases or biomarkers, among other applications [6]. There are two main possibilities [7]: inscribing the grating in the core of the fiber and depositing a thin film over the cladding, as in [8], where a silver-coated LPFG SPR sensor with a graphene monolayer is employed for detecting methane; or creating a nanostructure over the fiber, consisting of layers of different materials where a grating is manufactured, as in [9], where a structure with an Ag layer and a SiO_2_ or SiC grating for the detection of cortisol is studied.

D-shaped optical fibers offer different possibilities in order to manufacture optical sensors with optimized characteristics and performance, including the use of gratings [10]. In some cases, the D-shaped fiber is combined with another structure that includes a grating. This occurs in [11], where a D-shaped fiber is coated with a dielectric multilayer creating a one-dimensional photonic bandgap. The sensor is based on the Bloch surface wave (BSW) that occurs at the surface of the photonic band-gap and it achieves a sensitivity of 3.0 × 10^4^ dB/RIU in the 1.329–1.33 refractive index range. A D-shaped fiber combined with a TFBG is introduced in [12]. The D-shaped fiber is positioned in close proximity and parallel to the fiber containing the TFBG. Cladding modes from the TFBG are coupled to the D-shaped fiber with a power that depends on the SRI. Sensitivities from around 1000 to 13,000 nW/RIU are obtained in the SRI range from 1.33 to 1.45. In [13], an LPFG is presented that is patterned with laser micromachining techniques in a high-refractive-index overlay that has been deposited on the flat surface of a D-shaped optical fiber, which has been etched with hydrofluoric acid. The sensitivity is around 700 nm/RIU for SRI = 1.33.

However, there are other sensors in which the grating is directly written in the D-shaped optical fiber. In [14], part of the cladding of a D-shaped optical fiber is etched and the FBGs, which are known as surface-relief FBGs, are manufactured in the remaining cladding above the core. This device is capable of measuring temperature in the 20–120 °C range. Surface-relief gratings are also employed in [15], where they are combined with a polydimethylsiloxane (PDMS) layer in order to manufacture a volatile organic compound (VOC) sensor. The concentration of dichlorometane and acetone were each measured with a different sensor, achieving sensitivities of around 4000 ppm and around 6000 ppm, respectively. Finally, FBGs can be written in the core of D-shaped optical fibers by using laser point-by-point inscription with a sensitivity of around 30 nm/RIU for refractive indices around 1.45 [16].

In this article, a new structure consisting of FBGs inscribed in a thin film deposited on a D-shaped fiber is theoretically analyzed. The initial purpose of this study was to assess how FBG bands in the reflection spectrum are affected in magnitude, wavelength, and spectral width by the different parameters of the structure (thin-film thickness, cladding thickness between the core and the flat surface of the D-shaped fiber, and length). Then, the optimum was determined considering the magnitude of the FBG band, the full width half maximum (FWHM), the sensitivity to the surrounding refractive index (SRI), and the figure of merit (FOM).

The intended application for the proposed structure is biosensing, as optical fiber gratings (OFG) are being employed for chemical and biochemical sensing in label-free configurations due to their advantages, such as compactness, lightness, high compatibility with optoelectronic devices, and the possibility of multiplexing and remote sensing [17]. The proposed structure can also be utilized in the fabrication of distributed feedback (DFB) lasers, which are used in biosensing applications [18,19] due to their portable size, simple manufacture, and high sensitivity.

The article is organized as follows. In Section 2 a theoretical introduction is given and the simulated structure is presented. In Section 3, the results of the different simulations are explained. Finally, conclusions are presented in Section 4.

## 2. Theory

The structure under study consists of FBGs patterned on a SnO_2_ thin film that would be deposited on the flat surface of a D-shaped single mode optical fiber by using a mask. The grating pitch would be in the order of the µm with a 50% duty cycle (only half of each pitch is covered by the thin film). This way, the mask is feasible by employing an ultraviolet laser writer, which can have a resolution below the µm and it is thought to be repetitive enough for this step.

Once the mask has been fabricated, the rest of the process would consist in depositing the SnO_2_ thin film by sputtering [20] or atomic layer deposition [21], and finally removing the mask. There are also less expensive techniques such as layer-by-layer, which has been successfully used for SnO_2_ based lossy mode resonance (LMRs) sensors [22]. In the case of sputtering or atomic layer deposition, the corresponding equipment enables the fabrication of highly repetitive homogenous thin films, though a high degree of control in the parameters of layer-by-layer deposition (temperature, pH of the solutions, immersion times, drying times) should lead also to repeatable results.

**Figure 1:**
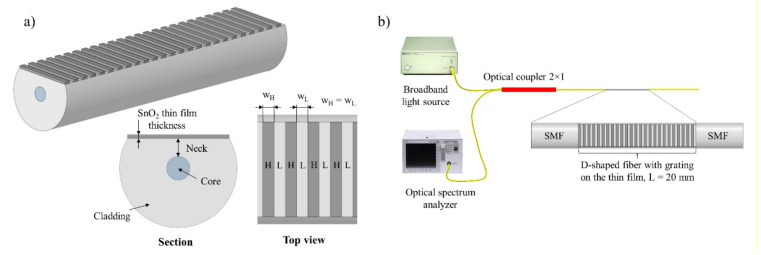
(**a**) A 3D schematic view of the grating on the flat surface of a D-shaped fiber with a close-up of the section and the grating on the lower part of the image. (**b**) The schematic reflection setup that is simulated. All dimensions in this figure are not to scale.

The structure’s main parameters and measuring setup, are shown respectively in Figure 1a,b. In Figure 1a, the neck is defined as the cladding thickness between the core and the flat surface of the D-shaped fiber, and is the term that will be used from here on.

The structures studied in this work were analyzed with the commercial software FIMMWAVE. The propagation was calculated with FIMMPROP, a module integrated with FIMMWAVE. For FBG sections, the finite difference method (FDM) with the Rigorous Coupled Mode Theory (RCMT) algorithm was employed because it enables efficient modeling of gratings.

The employed mesh is a uniform mesh (the chosen algorithm does not allow a nonuniform mesh) with 100 elements in the X direction and 1500 elements in the Y direction in the case of Section 3.1, for improving the accuracy. For the rest of the article, where it was observed that such precision was not required, the used mesh is 100 × 100. This mesh provides a reasonable trade-off between the precision of the results and the computational time, while it is too costly in terms of time to continue employing the previous mesh.

**Figure 2:**
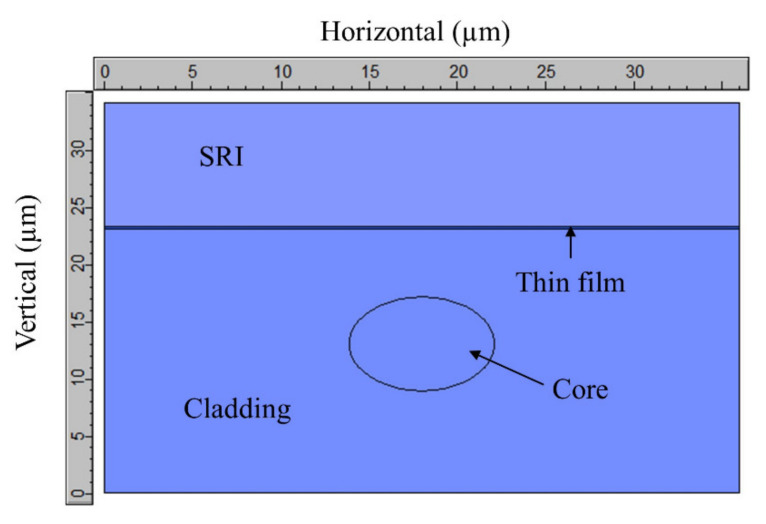
D-shaped fiber as modeled in FIMMWAVE.

The utilized section for modeling the D-shaped fiber is shown in Figure 2. The mesh is not shown as it would prevent us from distinguishing the shapes. As was demonstrated in [20], the results are equivalent to the ones obtained with the whole D-shaped section, but in this case computational time was saved. With respect to the boundary conditions, no perfectly matched layers (PML) were employed, while electric walls were placed at the left and right boundary conditions and magnetic walls were used at the top and bottom boundary conditions.

The work presented here is focused on the study of FBGs bands in the reflection spectrum, whose wavelength is calculated with (1), which has been adapted from the Bragg–Snell law for normal incidence [23]:

(1) $$m\lambda =2\left(n_{effH}w_{H}+n_{effL}w_{L}\right)$$

where *m* is the diffraction order and *n_effH_*, *n_effL_*, *w_H_*, and *w_L_* are the effective refractive indices and widths of the sections with the high- (H) and low- (L) refractive index materials respectively (see Figure 1a). 

In the structure under study, *n_effH_* and *n_effL_* correspond to the effective refractive index of the section of D-shaped fiber with and without thin film, respectively. Nevertheless, the SnO_2_ thin film does not appreciably affect the effective refractive index of the corresponding section. Therefore, it can be considered that *n_effH_* = *n_effL_* = *n_eff_*, where *n_eff_* is the effective refractive index of the structure. If it is taken into account that the addition of *w_H_* and *w_L_* is equal to the pitch of the grating, (1) can be simplified in our case to:

(2) $$m\lambda =2\cdot n_{eff}\cdot \Lambda$$

where Λ is the pitch of the grating.

**Figure 3:**
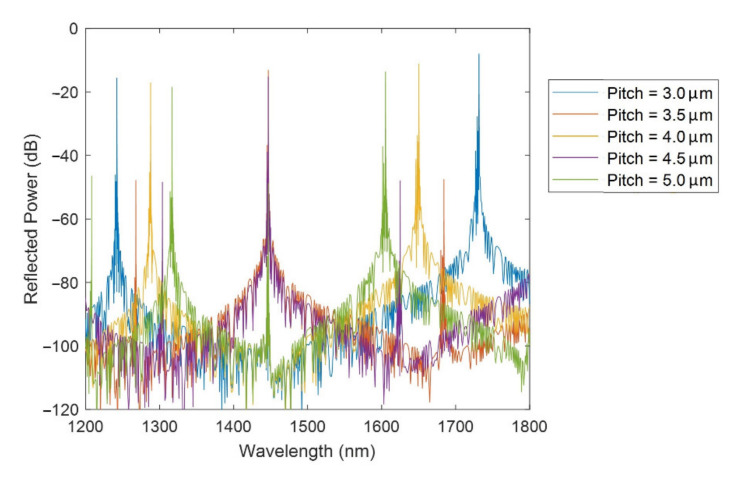
FBG bands in the 1200 - 1800 range for different pitches.

Figure 3 shows the FBG bands for different pitches: 3, 3.5, 4, 4.5, 5 µm. The simulations were carried out with these pitch values because lower pitches will increase the difficulty of manufacturing this structure, while higher pitches can lead to the appearance of other phenomena such as LMRs [24]. It can be appreciated that the reflected power of the FBG bands with odd *m* is higher than that of the FBG bands with even *m* (see the values of *m* in Table 1).

The length of the D-shaped zone was 20 mm (a typical length for a D-shaped fiber [20]), the SnO_2_ thin-film thickness was 125 nm and the neck was 4 µm. For the SnO_2_ thin film, a refractive index of 1.9 + 0.01i was used based on ellipsometric measurements in [25]. The analyzed optical spectrum ranges from 1200 to 1800 nm and the refractive index of the surrounding medium was 1.33 (water) as it was desirable to optimize this structure for its employment in biosensing applications. An SnO_2_ coated D-shaped fiber has been successfully used as a biosensor with a high degree of repeatability both for antiIgGs in human serum with femtomolar limit of detection [25], and also for D-dimer 5-fold below the clinical cutoff value of venous thromboembolism in human serum [26]. These results indicate that SnO_2_ is a stable material and the device proposed here should show a similar performance. It is also thought that the previously explained fabrication process would ensure the reproducibility of the device.

In the analyzed wavelength range (1200–1800 nm), the refractive index of fused silica is approximately 1.44 [27] and the effective refractive index of the core mode (*n_eff_*) is close to this value. The theoretical values for the position of the FBGs bands, obtained by substituting *n_eff_* = 1.44 in (2) and the different pitch values, are shown on the left side of Table 1. For the sake of clarity, only the points that fall in the studied range are shown. On the right side of Table 1, the values obtained from the simulations are included, showing that there is a high correspondence between those values and the ones calculated with (2). It can be remarked that, for a certain pitch, the lower the *m* value, the lower is the difference between the theoretical wavelength and the one obtained in the simulation.

**Figure 4:**
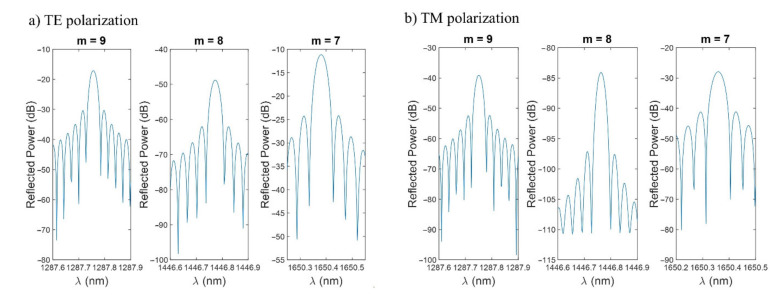
(**a**) Close-up of the FBG bands for a pitch of 4 µm and TE polarization in the 1200–1800 range. (**b**) Close-up of the FBG bands for a pitch of 4 µm and TM polarization in the 1200–1800 range.

In Figure 4, the three FBG bands in the 1200–1800 nm range for a pitch of 4 µm are shown in detail for both polarizations, with Figure 4a corresponding to transverse electric (TE) polarization and Figure 4b to transverse magnetic (TM) polarization. In this last case, the FBG bands are lower in power (around 20 dB for *m* = 7 and *m* = 9, and 35 dB for *m* = 8) and slightly blueshifted (the shift is between 5 and 20 pm and is not perceptible in Figure 4). Due to the better results obtained with TE polarization, the study is centered on this case, to which Figure 3 corresponds.

For the rest of the article, the simulations will be done with a pitch of 4 µm, TE polarization, and focusing on the FBG band located at 1650 nm as it is the most powerful one for the selected wavelength range and pitch. The study will focus on how FBG bands vary in power, wavelength, and FWHM by changing the different parameters of the structure (thickness of the thin film, neck, length). The optimum device will be selected considering the magnitude of the FBG band, the FWHM, the sensitivity to the SRI, and the FOM.

## 3. Results

First, the effects of the thin-film thickness and the neck on the reflected power, wavelength, and FWHM of the FBG bands were studied. Subsequently, the effect of varying the length of the D-shaped zone was analyzed. Finally, sensitivity to the SRI, FWHM, and FOM were assessed by modifying the SRI in the range 1.33–1.4 for a fixed thickness of the thin film and varying the neck, as well as the other way round. The study was focused on the FBG band centered at 1650 nm for a grating with a pitch of 4 µm and at TE polarization.

### 3.1. Study of the FBGs Bands for Variations of the Thin Film Thickness and the Neck

**Figure 5:**
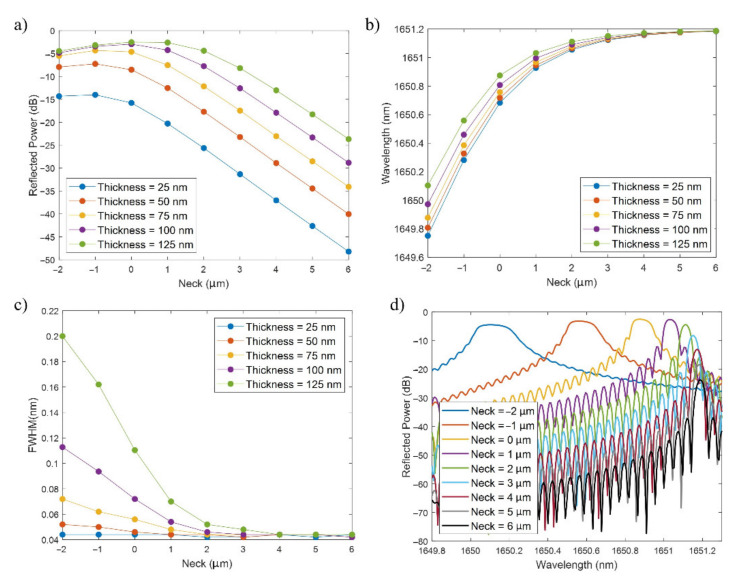
(**a**) FBG band reflected peak power (dB) versus neck (µm), (**b**) FBG band wavelength (nm) versus neck (µm), and (**c**) FBG band FWHM (nm) versus neck (µm), for different values of the thin film thickness. (**d**) FBG band for different necks and a thin-film thickness of 125 nm.

The analysis in this Subsection was carried out for SRI = 1.33 (water) and a length of the D-shaped zone of 20 mm (typical value). In Figure 5a, the peak power of the FBG band is shown for values of the neck in the range from −2 µm to −6 µm and thin-film thicknesses from 25 to 125 nm. 

Regarding the neck, negative values mean that part of the core has been etched. For instance, a neck of −1 µm implies that a thickness of 1 µm of the core has been etched. Lower (more negative) values of the neck are not studied because they produce large mode loss and the FBG peak is no longer recognizable in the reflection spectrum. Values of the neck higher than 6 µm are not assessed because the FBG band power decreases when the neck increases (see Figure 5a). In the case of the thin-film thickness, thicker values than 125 nm are not analyzed because the FWHM starts to become higher (values of several tenths of nm), especially for low necks, which decreases the FOM and, hence, the performance of the device as a sensor.

In Figure 5a, for a certain thin-film thickness, in general, the peak power of the FBG band slightly increases when the neck varies from −2 to −1 µm, it maintains a similar value for 0 µm and it clearly decreases in the range from −1 to 6 µm with a linear tendency of around −5 dB/µm. The maximum occurs for a neck of −1 μm or 0 µm, with power values that are between −2.5 dB and −4.5 dB for thicknesses between 75 and 125 nm, around −7 dB for a neck of 50 nm and −14 dB for a neck of 25 nm. This behavior can be explained by the fact that with a lower neck the effective index of the core is more influenced by the thin film, and as the neck increases, the perturbation is reduced and hence the FBG band is not so clearly visualized in the optical spectrum.

In addition, in the case of a fixed neck, the power increases with the thin-film thickness, which is again related to a higher perturbation in the effective index of the core mode with a thicker coating, closer to the mode transition region [28,29]. Here, two cases can be distinguished: for necks between −2 and 0 µm, the power variation as a function of thickness is not very relevant when the thickness is high (75–125 nm), whereas for positive values of the neck, the power difference among the different thicknesses tends to remain constant.

Regarding the central wavelength of the FBG band, it can be seen in Figure 5b that this parameter is sensitive to the D-shaped fiber neck as well as to the deposited SnO_2_ thickness. For a fixed thin-film thickness, the FBG band redshifts much more for negative necks (0.4–0.5 nm) than for higher values of the neck (few pm), where the wavelength converges to 1651.2 nm. This higher sensitivity for lower necks is explained by the higher interaction of the core mode with the outer medium, which leads to a reduction of the core mode effective index [30]. On the other hand, the redshift drops with the thickness, as can be observed by comparing the wavelengths for the different thicknesses for a neck of −2 µm and a neck of 6 µm, where all the wavelengths have converged to 1651.2 nm. This higher sensitivity for lesser thicknesses is explained by the countereffect produced by the mode transition phenomenon, which induces a higher increase in the core mode effective index as the coating thickness increases, reducing in this way the sensitivity induced by the reduction of the neck [28].

Another aspect that must be commented is the FWHM, which is shown in Figure 5c. The FWHM increases with the thickness, but decreases with the neck, converging to values of around 45 pm. For negative values of the neck and thicknesses between 100 and 125 nm, the FWHM values start to be higher than 100 pm, and they are even higher if the thickness is increased (points not shown). 

As the main conclusion of this section, the highest power is achieved for thicker thin films (100, 125 nm) and low necks (0, −1 µm), but these values lead to wider FWHM. This increase in the FWHM has also been observed in the case of FBGs, where the diameter of the cladding has been reduced [2], which is analogous to reducing the neck for the structure under study. In addition, it must be pointed out that, though greater thin-film thicknesses and lower necks have been avoided in order to prevent the FWHM from further broadening, the FWHM is much more affected by the SRI, an effect that will be studied in a posterior Subsection along with the sensitivity.

To end this section, the FBG bands for a thickness of 125 nm and different values or the neck are included in Figure 5d. This figure enables us to show some of the previously commented-on aspects. As the neck increases, the FBG band decreases in power, shifts towards the red (the shift is greater for the lower necks), and its FWHM is reduced.

### 3.2. Study of the FBGs Bands for Variations of the Length of the D-Shaped Zone

Until this point, the study has been carried out with a constant length of the D-shaped zone, 20 mm, a typical value for this parameter. Next, we studied the effect of varying the length of the D-shaped zone.

**Figure 6:**
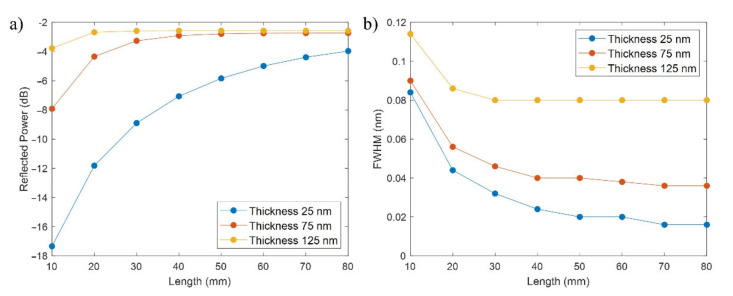
(**a**) FBG band reflected peak power (dB) vs. length (mm), and (**b**) FBG band FWHM (nm) vs. length (mm), for a neck of 0 µm and several thin-film thicknesses.

Simulations were carried out for thicknesses of 25, 75 and 125 nm with a neck of 0 µm and SRI = 1.33 (water). In Figure 6a, it is shown that the power increases with the length, quickly achieving convergence for a thickness of 125 nm (length of 30 mm), requiring a bit more in the case of 75 nm (50 mm) and still with some margin of improvement in the case of 25 nm for 80 mm. For all the thicknesses, it is notable that they tend to converge to a reflected power between −2.5 and −3 dB, although depending on the reflected power achieved for shorter lengths, they will require a smaller or greater increase in the length to converge. This agrees well with the conclusion obtained in Section 3.1, where thicker coatings led to FBG bands with higher reflected power because a higher perturbation was induced by a thicker coating, and with more periods, as is analyzed in Figure 6a, this lower reflected power observed with a thinner coating is progressively compensated.

On the other hand, in Figure 6b, it can be observed that the FWHM decreases with the length, achieving a more or less constant value for a length of 40 mm: 80 pm for a thickness of 125 nm, 40 pm for 75 nm, and 20 pm for 25 nm. In the case of the thicknesses of 75 and 25 nm, the FWHM still drops a bit while the length continues increasing. 

**Figure 7:**
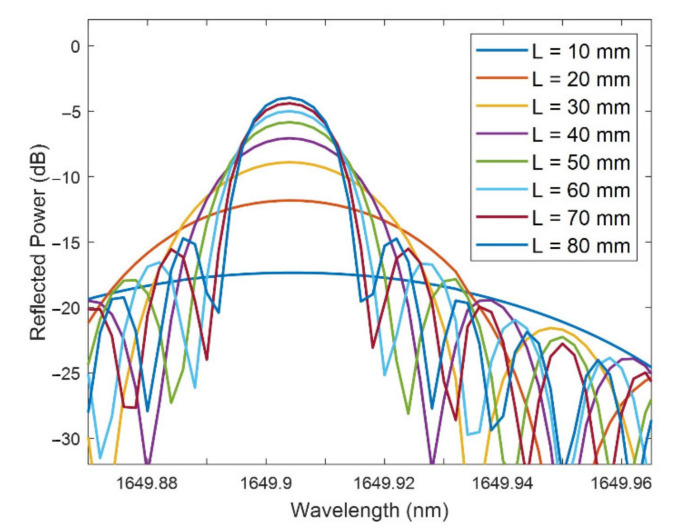
FBG bands for different values of the length of the D-shaped zone for a thin-film thickness of 25 nm and a neck of 0 µm.

The main conclusion of this Subsection is that the effect of increasing the length of the D-shaped zone is a rise in the reflected power of the FBG band and a decrease in the FWHM width. This can also be observed in Figure 7, where the FBG bands are shown for a thickness of 25 nm, neck of 0 µm, and lengths from 10 to 80 mm. However, it has been previously observed that a point is always reached where the power ceases increasing and the FWHM stops reducing. Finally, another aspect worth mentioning in Figure 7, which had not appeared until now, is that the central wavelength of the FBG band is not sensitive to the length value.

### 3.3. Study of the FBGs Bands for Variations of the SRI

First, the sensitivity to the SRI and the FOM of the FBG bands have been studied for a variation of the surrounding medium refractive index (SRI) between 1.33 and 1.4 for necks of −2, 0, and 2 µm. The D-shaped zone length was 20 mm and the thickness of the SnO_2_ thin film was fixed to 125 nm, a value that was selected because it leads to the FBG bands with the highest reflected power among the studied thicknesses. 

**Figure 8:**
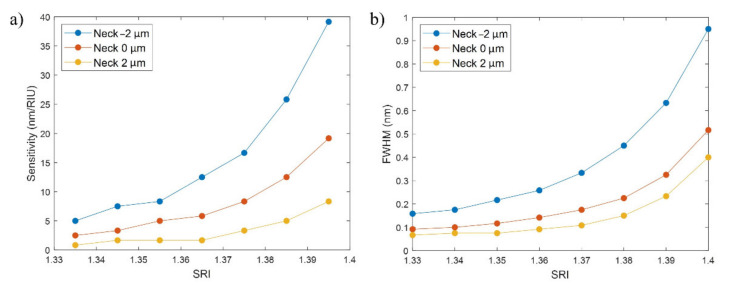
(**a**) Sensitivity to the SRI (nm/RIU) vs. SRI (RIU), and (**b**) FWHM (nm) vs. SRI (RIU), for a thin-film thickness of 125 nm and necks of −2, 0, and 2 µm.

In Figure 8a the sensitivity increases with the value of the SRI and when the neck is thinner. This is coherent with the fact that FBGs have a greater sensitivity when the cladding diameter reduces [2], which happens because the effective index of the core is more affected by the external medium as the core is closer to it.

Each point in the graph represents the sensitivity between the two refractive indices that are immediately below and above that point. It can be observed that for low values of the SRI, the sensitivity is around or below 5 nm/RIU. In the case of the highest SRI, the values achieved are in the 5–10 nm/RIU range for a neck of 2 µm, 10–20 nm/RIU for 0 µm, and 15–40 nm/RIU for −2 µm.

As previously happened with the power, the main drawback of the sensitivity is that when it increases, the FWHM grows, achieving values of several tenths of nm for sensitivities that are above 10 nm/RIU if the corresponding points from Figure 8a,b are compared.

In order to condense the information regarding the sensitivity to the SRI and the FWHM, the figure of merit (FOM) was calculated for SRI = 1.34 and SRI = 1.40 (see Table 2). The lowest FOM values correspond to a neck of 2 µm, with values achieving at most 26.31 RIU^−1^ because, although the FWHM values are lower than for the rest of the neck values, the sensitivities are much lower. For a neck of 0 µm, the FOM increases from 26.08 to 45.54 RIU^−1^ between SRI = 1.34 and SRI = 1.40, while for a neck of −2 µm the FOM increases from 30.00 to 49.47 RIU^−1^. The FOM values are similar because the higher sensitivities for a neck of −2 µm are compensated by narrower FWHMs in the case of the neck of 0 μm, although they are slightly better in the case of −2 μm.

Consequently, both a thin-film thickness of 125 nm and a neck of −2 μm or 0 μm constitute a good parameter selection to implement a real device, as they have similar high FOMs and high power, as was seen in the first Subsection of the results. The final choice between the neck of−2 μm or 0 μm depends on whether the sensitivity (higher for −2 μm) or the FWHM (narrower for 0 μm) is prioritized.

**Figure 9:**
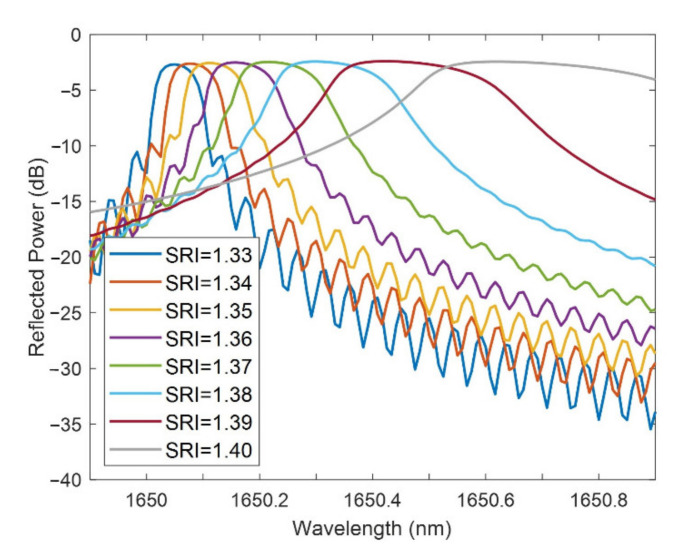
FBG band for different SRI for a thin-film thickness of 125 nm and a neck of 0 µm.

In Figure 9, the FBG bands are shown for a thin-film thickness of 125 nm and a neck of 0 µm for SRI ranging from 1.33 and 1.4. It is clear, as has been previously described, that the sensitivity rises with the SRI (the redshift in Figure 9 increases with the SRI), but the FWHM widens. The reflected power does not significantly change.

Finally, to end this study, the effect of reducing the thin-film thickness was analyzed. From previous results, diminishing the thickness will lower the power and the FWHM but it is still unknown what will occur with the sensitivity and the FOM.

In this last part of the analysis, the sensitivity of the FBG bands and the FOM were analyzed for a variation of the SRI between 1.33 and 1.4 for a neck of −2 µm and thicknesses of 25, 75, and 125 nm, while the length of the D-shaped zone was fixed to 20 mm.

**Figure 10:**
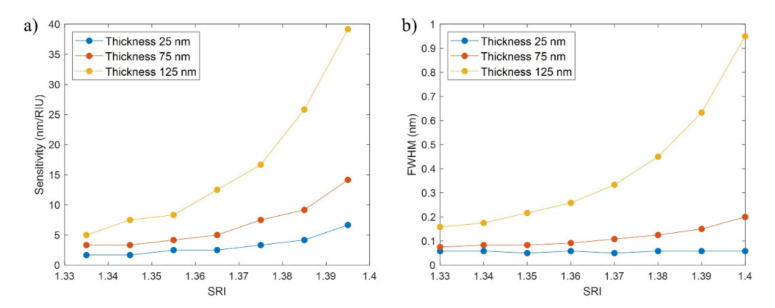
(**a**) Sensitivity to the SRI (nm/RIU) vs. SRI (RIU), and (**b**) FWHM (nm) vs. SRI (RIU), for a neck of −2 µm and thin-film thicknesses of 25, 75, and 125 nm.

It can be observed in Figure 10a that the sensitivity increases as a function of the SRI and the thin-film thickness, an effect that is related to the higher proximity to the mode transition region when the thin film is thicker [28]. For a thickness of 25 nm, the sensitivity is below 5 nm/RIU for most of the points, achieving a value of 6.67 only between SRI of 1.39 and 1.4. In the case of the 75 nm thickness, for an SRI between 1.33 and 1.37, the sensitivity is below 5 nm/RIU, while values between 5 and 15 nm/RIU are achieved between SRI of 1.38 and 1.4. With a thickness of 125 nm the highest sensitivities are obtained, with values between 5 and 10 nm/RIU between SRI of 1.33 and 1.36 that quickly rise for higher SRI, even reaching values of 39 nm/RIU.

Again, the weakest point in attaining a high sensitivity is that the FWHM of the corresponding FBG band widens, as can be seen in Figure 10b. Nevertheless, in the case of the 75 nm thickness, the sensitivities are between 3 and 15 nm/RIU for FWHM between 70 and 200 pm, which can be considered an adequate trade-off between sensitivity and FWHM.

To summarize the information provided by the sensitivity to the SRI and the FWHM, the FOM is calculated for SRI = 1.34 and SRI = 1.40 (see Table 3). In this case, the FWHM has more influence in the comparison between the results for the different thicknesses. The best FOM values are achieved for a thickness of 25 nm due to their very low FWHMs, varying from 28.57 to 114.29 RIU^−1^ between SRI = 1.34 and SRI = 1.40. In the case of the thickness of 75 nm, the FOM increases for 42.10 to 80.95 RIU^−1^ in this range, while for 125 nm it varies from 30.00 to 49.47 RIU^−1^ as has been previously seen in Table 2.

Therefore, it can be concluded that the FOM increases when the thin film is reduced. Consequently, doubt may arise as to whether a thickness of 125 nm and a neck of −2 μm constitute a good choice of parameters, as was previously mentioned, or whether it is better to reduce the thickness. It has to be considered that greater FOMs for low thicknesses are achieved thanks to lower FWHMs, while the sensitivities are much lower. As a result, a trade-off solution would be choosing a thickness of 75 nm with a neck of −2 μm. The sensitivities are not the highest ones (between 3 and 15 nm/RIU), but the power is high (see again Figure 5a) and the FOM is high (between 40 and 80 RIU^−1^). 

**Figure 11:**
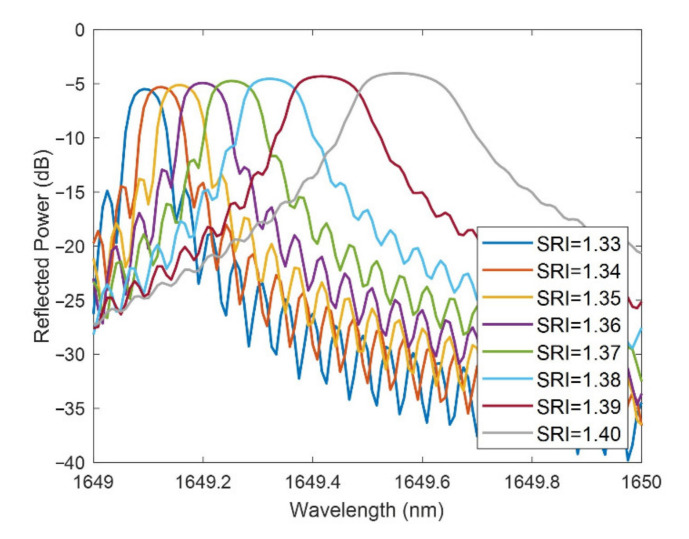
FBG band for different SRI for a neck of −2 µm and a thin-film thickness of 75 nm.

In Figure 11, the FBG bands are shown for a neck of −2 µm and a thin-film thickness of 75 nm with SRI between 1.33 and 1.4. The sensitivity rises with the SRI (the redshift in Figure 11 increases with the SRI) but the FWHM broadens.

The first conclusion of this section is that the sensitivity is favored by a thicker thin film, a low (negative) neck and a high SRI; but a higher sensitivity leads to a wider FWHM. It is not difficult to reach sensitivities between 5–10 nm/RIU and even higher if the different parameters are properly combined to achieve the optimum. However, the higher the sensitivity, the wider the FWHM—easily achieving values of several tenths of nm for sensitivities above 10 nm/RIU.

Regarding the FOM, it increases with a low (negative) neck and reduces with the thin-film thickness. High values (between 40 and 80 RIU^−1^) are obtained but they require a high sensitivity (which will have the disadvantage of a wide FWHM) or a narrow FWHM (which will present, as a drawback, a low sensitivity). 

Comparing the results for the structure under analysis obtained in this section with the FBG-based sensors studied in [2], in the case of the maximum sensitivity (39 nm/RIU), it would be an acceptable value in spite of being far from the top sensitivities, which can reach values of hundreds or even thousands of nm/RIU. Regarding the FOM, values between 40 and 80 RIU^−1^ (the highest one was 114.29 RIU^−1^) are quite high, only being surpassed by very few sensors from [2]. In the case of the FWHM, the lowest values, around 45 pm, are better than those attained with any FBG-based sensor in [2]. It has to be considered that not all the parameters (sensitivity, FOM, FWHM) of the structure can be optimized at the same time, so some of them will have to be prioritized.

## 4. Conclusions

A new structure consisting of a fiber Bragg grating patterned on a SnO_2_ thin film deposited on the flat surface of a D-shaped optical fiber was analyzed as a function of several design parameters.

In this first place, it was demonstrated that the position of the FBG bands for different pitches can be predicted with accuracy by adapting the Bragg–Snell law for normal incidence.

Then, the study was focused on the FBG band located at 1650 nm for a grating with a pitch of 4 µm and SRI = 1.33 (water). The reflected power and the FWHM were studied for variations in the thin-film thickness, the neck (cladding thickness between the nucleus of the cladding and the flat surface of the D-shaped fiber) and the length. The first conclusion is that peak powers between −2.5 and −8.5 dB can be achieved for thicknesses in the 50–125 nm range with necks between −2 and 0 µm. Increasing the reflected power comes at the cost of widening the FWHM, which is in the order of 100 pm. Regarding the length, increasing its value improves both the reflected power and the FWHM, although in practice it is not realistic to work with lengths much greater than 20 mm.

Then, the sensitivity to the SRI, the FWHM and the FOM were studied for variations in the SRI in the 1.33–1.4 range. From the first part of the analysis, it seemed that the best choice for implementing the grating under study was a thicker thin-film and a low (negative) neck (125 nm and −2, −0 µm respectively). However, for these cases, the sensitivity is high (values between 10 and 40 nm/RIU for the highest SRI), at the cost of penalizing the FWHM (several hundreds of pm) and therefore the FOM, which is in the 30−50 RIU^−1^ range. Nevertheless, thinner thin films, while maintaining a low neck, will reduce the sensitivity but they will increase the FOM. For a 75 nm thin-film thickness and −2 µm neck, the sensitivity to the SRI is in the 3–15 nm/RIU range (a bit low), but the FWHM does not surpass 200 pm and the FOM can reach values of 80 RIU^−1^.

The final selection of parameters for implementing this structure will depend on whether the sensitivity to the SRI or the FOM is prioritized, but it seems the optimum design will require an intermediate–high thin-film thickness among the studied range (75 to 125 nm) and a low, even negative value for the neck (−2 to −0 µm). The effect of varying the refractive index and the extinction coefficient of the thin film material could also be explored, although it is not under the scope of this text.

As a conclusion, the generation of gratings on thin films on D-shaped fibers opens a new path for optical fiber sensors based on this technology. In addition to this, this structure benefits from the use of thin films that can be already sensitive to a parameter, enabling the development of environmental, gas, chemical sensors, or biosensors.

## Figures and Tables

**Table 1:** Theoretical (left) and simulated (right) wavelengths for the FBG bands.

	Theoretical Wavelengths (nm)					Simulated Wavelengths (nm)				
m	Pitch 3.0 µm	Pitch 3.5 µm	Pitch 4.0 µm	Pitch 4.5 µm	Pitch 5.0 µm	Pitch 3.0 µm	Pitch 3.5 µm	Pitch 4.0 µm	Pitch 4.5 µm	Pitch 5.0 µm
5	1728					1731.54				
6	1440	1680				1446.76	1684.22			
7	1234	1440	1646			1242.23	1446.77	1650.39		
8		1260	1440	1620	1800		1267.84	1446.77	1624.99	1802.40
9			1280	1440	1600			1287.76	1446.77	1605.21
10				1296	1440				1303.69	1446.78
11					1309					1316.72
12					1200					1208.06

**Table 2:** FOM (RIU^−1^) for a thin-film thickness of 125 nm.

SRI	Neck −2 µm	Neck 0 µm	Neck 2 µm
1.34	30.00	26.08	11.76
1.40	49.47	45.54	26.31

**Table 3:** FOM (RIU^−1^) for a neck of −2 µm.

SRI	Thin Film Thickness		
	25 nm	75 nm	125 nm
1.34	28.57	42.10	30.00
1.40	114.29	80.95	49.47

## Data Availability

The data that support the findings of this study are available from the corresponding authors upon request.
